# Expression of peroxisome proliferator-activated receptor (PPAR)**γ** in gastric cancer and inhibitory effects of PPAR**γ** agonists

**DOI:** 10.1054/bjoc.2000.1457

**Published:** 2000-10-26

**Authors:** H Sato, S Ishihara, K Kawashima, N Moriyama, H Suetsugu, H Kazumori, T Okuyama, M A K Rumi, R Fukuda, N Nagasue, Y Kinoshita

**Affiliations:** Second Department of Internal MedicineSecond Department of Surgery, Shimane Medical University, Izumo, Shimane, Japan

**Keywords:** PPARγ, gastric cancer, growth inhibition, apoptosis

## Abstract

Peroxisome proliferator-activated receptor (PPAR) γ is expressed in human colon cancer, prostate cancer and breast cancer cells, and PPARγ activation induces growth inhibition in these cells. PPARγ expression in human gastric cancer cells, however, has not been fully investigated. We report the PPARγ expression in human gastric cancer, and the effect of PPARγ ligands on proliferation of gastric carcinoma cell lines. Immunohistochemistry was used to demonstrate the presence of PPARγ protein in surgically resected specimens from well differentiated, moderately differentiated and poorly differentiated adenocarcinoma. We used reverse transcription-polymerase chain reaction and Northern and Western blot analyses to demonstrate PPARγ expression in four human gastric cancer cell lines. PPARγ agonists (troglitazone and 15-deoxy-Δ^12,14^-prostaglandin J2) showed dose-dependent inhibitory effects on the proliferation of the gastric cancer cells, and their effect was augmented by the simultaneous addition of 9-*cis* retinoic acid, a ligand of RXRα. Flow cytometry demonstrated G1 cell cycle arrest and a significant increase of annexin V-positive cells after treatment with troglitazone. These results suggest that induction of apoptosis together with G1 cell cycle arrest may be one of the mechanisms of the antiproliferative effect of PPARγ activation in human gastric cancer cells. © 2000 Cancer ResearchCampaign


*British Journal of Cancer* (2000) **83**(10), 1394–1400
© 2000 Cancer Research Campaign
doi: 10.1054/ bjoc.2000.1457, available online at http://www.idealibrary.com on IDEAL®

### Expression of peroxisome proliferator-activated receptor (PPAR)γ in gastric cancer and inhibitory effects of PPARγ agonists

**H Sato[1], S Ishihara[1], K Kawashima[1], N Moriyama[1], H Suetsugu[1], H Kazumori[1], T Okuyama[1], MAK Rumi[1], R Fukuda[1], N Nagasue[2] and Y Kinoshita[1]**

[1]Second Department of Internal Medicine; [2]Second Department of Surgery, Shimane Medical University, Izumo, Shimane, Japan

**Summary** Peroxisome proliferator-activated receptor (PPAR) γ is expressed in human colon cancer, prostate cancer and breast cancer cells, and PPARγ activation induces growth inhibition in these cells. PPARγ expression in human gastric cancer cells, however, has not been fully investigated. We report the PPARγ expression in human gastric cancer, and the effect of PPARγ ligands on proliferation of gastric carcinoma cell lines. Immunohistochemistry was used to demonstrate the presence of PPARγ protein in surgically resected specimens from well differentiated, moderately differentiated and poorly differentiated adenocarcinoma. We used reverse transcription-polymerase chain reaction and Northern and Western blot analyses to demonstrate PPARγ expression in four human gastric cancer cell lines. PPARγ agonists (troglitazone and 15-deoxy-$\Delta^{12,14}$-prostaglandin J2) showed dose-dependent inhibitory effects on the proliferation of the gastric cancer cells, and their effect was augmented by the simultaneous addition of 9-*cis* retinoic acid, a ligand of RXRα. Flow cytometry demonstrated G1 cell cycle arrest and a significant increase of annexin V-positive cells after treatment with troglitazone. These results suggest that induction of apoptosis together with G1 cell cycle arrest may be one of the mechanisms of the antiproliferative effect of PPARγ activation in human gastric cancer cells. © 2000 Cancer Research Campaign

**Keywords:** PPARγ; gastric cancer; growth inhibition; apoptosis

Thiazolidinediones, including troglitazone, and 15-deoxy-$\Delta^{12,14}$-prostaglandin J2 (15d-PGJ2), a metabolite of prostaglandin J, have been identified as ligands for peroxisome proliferator-activated receptor (PPAR) γ (Forman et al, 1995; Kliewer et al, 1995). PPARγ has been reported to play an important role in insulin sensitization and adipocyte (Chawla et al, 1994) and monocyte/macrophage (Tontonoz et al, 1998; Pelton et al, 1999) differentiation. Expression and activation of PPARγ in fibroblastic and myoblastic cells triggers the adipocyte gene expression cascade and leads to the development of the adipose phenotype (Tontonoz et al, 1994; Hu et al, 1995). This receptor and its heterodimeric partner, retinoid X receptor (RXR) α, which binds to 9-*cis* retinoic acid (9-cis RA), form a DNA-binding complex. Transcriptional activity of the PPARγ/RXRα heterodimer is maximal in the presence of both PPARγ and RXRα activators (Mukherjee et al, 1997). Recent reports have indicated that PPARγ also express in different tissues and PPARγ ligands can induce growth inhibition in human prostate cancer cells (Kubota et al, 1998), colon cancer cells (Brockman et al, 1998; Sarraf et al, 1998; Kitamura et al, 1999) and liposarcoma cells (Tontonoz et al, 1997). Induction of apoptosis by PPARγ has been demonstrated in mammalian cells (Elstner et al, 1998; Clay et al, 1999; Keelan et al, 1999). A recent study has demonstrated expression of PPARγ in human gastric cancer cell line MKN45 and reported troglitazone-induced growth inhibition and apoptosis (Takahashi et al, 1999). To evaluate the possibility of PPARγ ligands in gastric cancer treatment, in the present study we have investigated PPARγ expression in human gastric carcinoma tissues and checked growth inhibitory effects of different types of PPARγ agonists on four cell lines.

*Received 15 February 2000*
*Revised 10 July 2000*
*Accepted 12 July 2000*

*Correspondence to*: H Sato

### MATERIALS AND METHODS

### Immunohistochemistry for PPARγ protein

Human gastric cancer tissues were obtained at the time of surgical removal with the informed consent of the patients. The corresponding normal gastric tissues were also obtained simultaneously. These samples were immediately frozen and stored in liquid nitrogen until immunohistochemistry. Identification of cancer and non-cancer specimens was confirmed by light microscopic examination. From these samples, 3-μm thick cryostat tissue sections were mounted on poly-L-lysine-coated slides and stored at –20°C. The slides were air-dried for 30 min and fixed in acetone at –4°C for 10 min. Samples were then incubated with normal horse serum (Vector Laboratories, Burlingame, CA, USA) for 30 min, followed by incubation with a mouse monoclonal antibody recognizing human PPARγ (Santa Cruz Biotechnology, Santa Cruz, CA, USA) for 120 min. Mouse IgG1 myeloma protein MOPC-21 (Sigma, St Louis, MO, USA) was used as control. After washing the sections with phosphate buffered saline (PBS), they were incubated with biotinylated horse anti-mouse IgG (Vector Laboratories) for 30 min. Bound antibody was detected using the avidin–biotin peroxidase method (ABC Kit; Vector Laboratories). Peroxidase activity was subsequently detected by 3,3′-diaminobenzidine (Sigma) in 0.05 M Tris-HCl for 10 min at room temperature, followed by haematoxylin staining. After dehydration in a graded alcohol series, the sections were cleared in xylene.

### Cell lines

Four human gastric cancer cell lines, MKN-7, MKN-28, MKN-45 and AGS were used in this study. MKN-7, MKN-28 and MKN-45 were obtained from Riken Cell Bank (Ibaraki, Japan) and AGS from the American Type Culture Collection (ATCC, Rockville, MD, USA). The MKN-45 and AGS were established from poorly differentiated gastric carcinoma and MKN-7 and MKN-28 from well and moderately differentiated gastric carcinoma. MKN-7, MKN-28 and MKN-45 cells were cultured in RPMI 1640 (ICN Biomedicals, Ohio, USA) and AGS cells in Ham's F12 (ICN) supplemented with 10% fetal bovine serum in an atmosphere of 5% $CO_2$ at 37°C in a humidified incubator.

### RNA extraction

Total RNA was extracted by the acid guanidinium phenol chloroform method using Isogen (Nippon Gene, Tokyo, Japan). $5 \times 10^6$ cells were shaken vigorously for 1 min in 0.8 ml of Isogen solution and 0.2 ml of chloroform. After centrifugation at 12 000 rpm at 4°C for 15 min, the aqueous phase was transferred to a fresh tube, an equal volume of isopropanol was added and the mixture was allowed to stand at 4°C for 15 min, followed by centrifugation at 12 000 rpm at 4°C for 15 min. The RNA precipitate was rinsed with 75% ethanol and air-dried for 5 min. Finally, RNA was dissolved in 50 μl of distilled water containing 0.1% diethylpyrocarbonate and the RNA concentration was measured by spectrophotometry at 260 nm.

### Reverse transcription-polymerase chain reaction

Five μg total RNA extracted from each cell line was reverse transcribed to cDNA using a First Strand cDNA Synthesis Kit (Stratagene, Toyobo, Japan) according to the manufacturer's instructions using oligo (dT) primer. Synthesized cDNA was stored at –80°C until used for the polymerase chain reaction assay (PCR). The primers used for amplifying PPARγ cDNA were 5′-TCTGGCCCACCAACTTTGGG-3′ and 5′-CTTC-ACAAGCATGAACTCCA-3′ and for RXRα cDNA were 5′-CTCTCAGGTTGAACTCACCT-3′ and 5′-ATCTCTGACAG-CCTGTCTCG-3′. As internal control, RT-PCR for β-actin mRNA was also performed using the primers (5′-ATCTGGCACCA-CACCTTCTACAATGAGCTGCG-3′ and 5′-CGTCATACTCCT-GCTTGCTGATCCACATCTGC-3′). After denaturation of the samples at 95°C for 10 min, PCR was carried out in a DNA thermal cycler (PE Biosystems, Foster City, CA, USA) for 35 cycles (95°C for 1 min, 55°C for 30 s and 72°C for 1 min) followed by 72°C for 10 min. 5 μl of PCR products were electrophoresed in 1% agarose gels stained with ethidium bromide. The detection of amplified DNA bands at the expected lengths (PPARγ 360 bp, RXRα 422 bp, β-actin 838 bp) was confirmed and products were also directly sequenced by ABI Prism 310 Genetic Analyser using BigDye terminator Cycle Sequencing Reagent (PE Biosystems).

### Northern blot analysis

From each cell line, 20 μg total RNA was electrophoresed in formaldehyde-containing 1.2% agarose gels and transferred to a nitrocellulose membrane (Schleicher and Schuell, Dassel, Germany), followed by fixation with UV cross-linker (Funa-UV-Linker; Funakoshi, Tokyo, Japan). The probes used for Northern blot analysis were a $^{32}$P-labelled 360 bp cDNA fragment of human PPARγ and a $^{32}$P-labelled 838 bp cDNA fragment of human β-actin. After 4 h prehybridization and 24 h hybridization at 42°C, the filters were washed first for 10 min and then for 15 min at 37°C in 2 × saline-sodium citrate (SSC) containing 0.2% sodium dodecyl sulphate (SDS), and finally at 50°C in 0.1 × SSC containing 0.2% SDS for 20 min. Autoradiography was performed using image analyser BAS 2000 II (Fuji Photo Film Co, Tokyo, Japan).

### Western blot analysis

Protein concentrations were measured using Bio-Rad Protein Assay Reagent (Bio-Rad Laboratories, Richmond, CA, USA) following the manufacturer's instruction. 50 μg of protein was separated by 10% SDS PAGE. After electrophoresis, the proteins were transferred to polyvinylidene membranes (Amersham International, Buckingham, UK), blocked in TBS-Tween with 10% skimmed milk at 4°C for 1 h, subsequently reacted with primary monoclonal antibody overnight and washed. After reacting with a mouse peroxidase-conjugated antibody detection agent (Amersham), signal was detected by chemiluminescence using the ECL detection system (Amersham). For detection of PPARγ, mouse anti-human PPARγ monoclonal antibody (SC-7273, Santa Cruz) was used. As an internal control, β-actin was detected with mouse monoclonal antibody (AC-15, Sigma).

### Growth assessment and chemicals

Human gastric cancer cells were seeded ($1 \times 10^5$ cells ml$^{-1}$) in 24-well plates and treated for 48 h with each chemical agent. Troglitazone was kindly provided by Sankyo Pharmaceutical Co (Tokyo, Japan) and dissolved in dimethyl sulphoxide (DMSO). Indomethacin (Nacalai tasque, Kyoto, Japan) was also dissolved in DMSO. 9-cis RA (Sigma) and 15d-PGJ2 (Calbiochem, La Jolla, CA, USA) were dissolved in ethanol. Vehicle in the medium did not exceed 0.1%. After 48 h treatment with drug, 1 μCi [Methyl-$^3$H] thymidine (Amersham) was added to each well and incubated for 4 h. Cells were harvested onto a glass fibre filter mat using a Cell Harvester (Inotech, Switzerland). After the mat was dried [$^3$H] thymidine incorporation was measured by 1450 Microbeta scintillation counter (Wallac, Oy, Turku, Finland). Assessment of cell proliferation was performed in triplicate and repeated three times.

### Cell cycle analysis

Cell cycle profiles were performed on all four gastric cancer cell lines. Cells were treated with 10 μM troglitazone for 48 h, collected after brief trypsinization, washed with PBS, and fixed in cold 70% ethanol. Then the samples were treated with RNase, stained with 50 μg ml$^{-1}$ propidium iodide (PI), and analysed by EPICS Elite flow cytometer (Coulter Electronics, FL, USA).

© 2000 Cancer Research Campaign

### Detection of apoptosis in gastric cancer cells

To detect apoptotic cells, the expression of annexin V on the cell surface was examined by flow cytometry. Annexin V detects phosphatidylserine on the outer cytoplasmic membrane, which occurs during the loss of phospholipid asymmetry early in the apoptotic process. After 48 h treatment with troglitazone, the supernatant was removed and the adherent cells were harvested with 0.05% trypsin. The cells were washed three times with PBS and incubated with FITC-conjugated annexin V antibody (Immunotech, Marseille, France) and PI in medium containing 1.5 mM $CaCl_2$ at 4°C for 15 min. After incubation, cells were analysed by flow cytometry (FACScan; Becton Dickinson, San Jose, CA, USA). The assay was done in triplicate and repeated three times. PI-negative and annexin V-positive cells were considered as early apoptotic cells.

Nuclear morphology of apoptosis was assessed by staining with Hoechst 33258 (Calbiochem); cells with condensed or fragmented nuclei were recognized as apoptotic cells by fluorescence microscopy (Olympus, Tokyo, Japan).

### Statistical analysis

Data are expressed as means ± SE. Values were compared and significant differences between means were determined by Analysis of variance (ANOVA). Multiple comparisons were done by Sheff's test after ANOVA and $P$ values < 0.05 were considered statistically significant.

### RESULTS

### Expression of PPARγ protein in human gastric cancer

Surgically obtained tissue from gastric adenocarcinoma expressed PPARγ protein, as shown by immunohistochemistry (Figure 1). We stained sections from 12 selected cases – four poorly differentiated, four moderately differentiated and four poorly differentiated cancers. The staining result was consistent in all cases. PPARγ protein was expressed not only in well differentiated, moderately differentiated and poorly differentiated gastric adenocarcinoma, but also normal mucosa with intestinal metaplasia adjacent to cancer.

### PPARγ and RXRα expression in human gastric cancer cell lines

We used RT-PCR to determine the expression of PPARγ and RXRα mRNA in four gastric cancer cell lines: MKN-7, MKN-28, MKN-45 and AGS. As shown in Figure 2A, PPARγ and RXRα mRNA were expressed in all the four cell lines. PPARγ mRNA was expressed at relatively higher levels in MKN-7 and MKN-45 cells (Figure 2B). PPARγ protein was also detected in all four cell lines, but only at a low level in AGS cells. As an internal control, we demonstrated β actin expression (Figure 2C).

### Growth assessment of human gastric cancer cell lines

We evaluated the effect of various ligands on the proliferation of the four gastric cancer cell lines by [$^3$H]-thymidine incorporation. There was a dose-dependent reduction in [$^3$H]-thymidine uptake after treatment with troglitazone for 48 h in three cell lines,

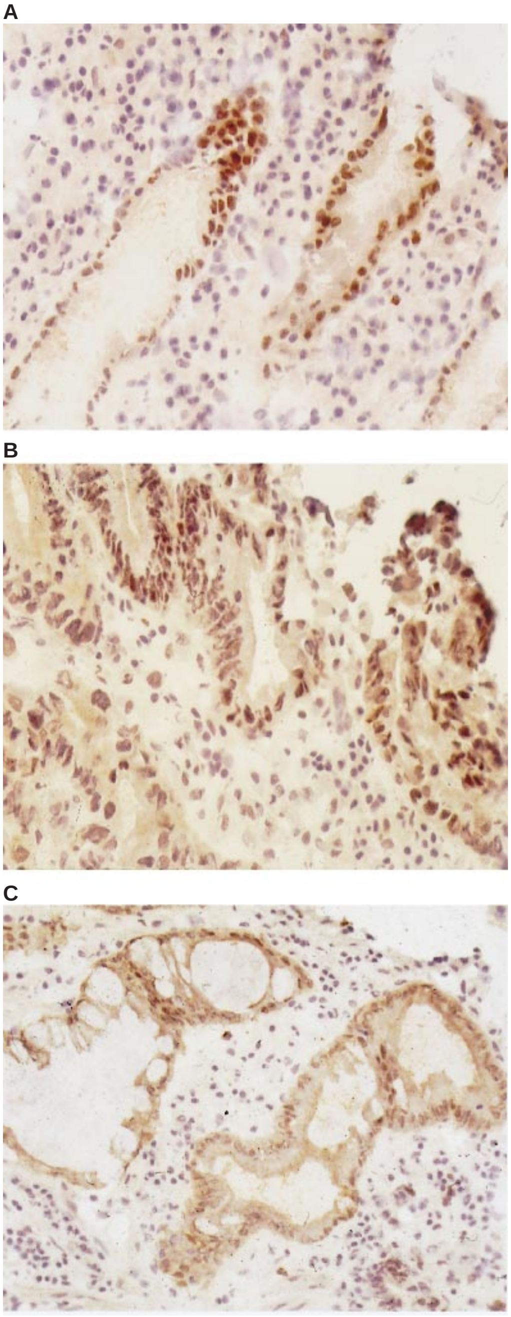

**Figure 1** PPARγ protein expression in human gastric cancer tissues. PPARγ protein is expressed in both well differentiated (**A**) and poorly differentiated (**B**) adenocarcinoma. In addition, PPARγ protein is also present in non-cancerous tissue with intestinal metaplasia (**C**) adjacent to cancer tissue

MKN-28, MKN-45, and AGS. Troglitazone induced a significant ($P$ < 0.05) antiproliferative effect at a concentration of 10 μM in these cell lines. Treatment with 15d-PGJ2, a natural ligand for PPARγ, also reduced [$^3$H]-thymidine uptake in MKN-45 and AGS cells. However, in MKN-7 cells, increased incorporation of [$^3$H]-thymidine was observed after treatment with troglitazone or 15d-PGJ2. Indomethacin, a ligand for PPARγ (Lehmann et al, 1997), and 9-cis RA, a ligand for RXRα, showed weak growth suppression at high concentrations (Figure 3), but their effects were

 © 2000 Cancer Research Campaign

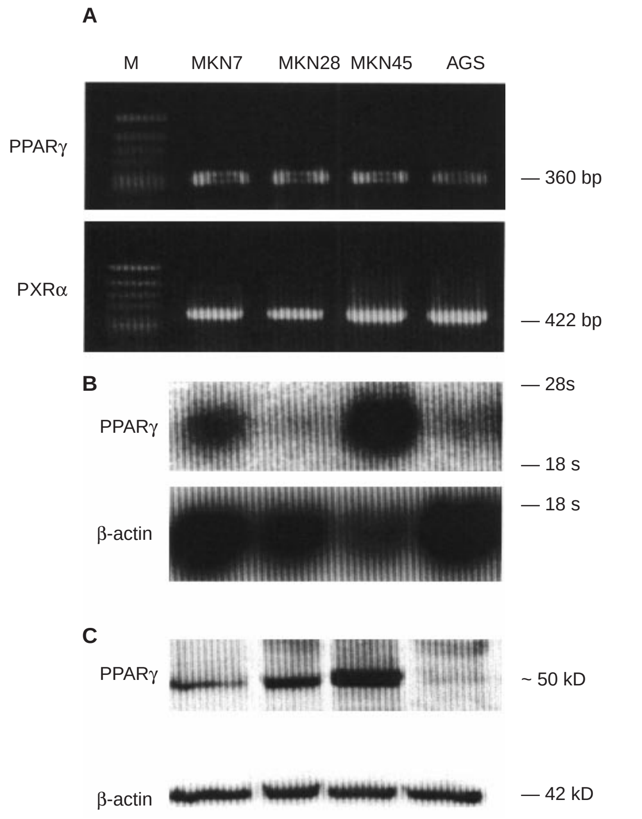

**Figure 2** PPARγ expression in human gastric cancer cell lines. (**A**) PPARγ and RXRα expression at the mRNA level in human gastric cancer cell lines. From each cell line, 5 μg of total RNA was subjected to RT-PCR. 5 μl aliquot of the PCR products were electrophoresed in 1% agarose gel. (**B**) Northern blot analysis of PPARγ expression in human gastric cancer cell lines. Approximately 20 μg of RNA of each cell line was electrophoresed through formaldehyde-containing 1.2% agarose gels and transferred to a nitrocellulose membrane. Hybridization was performed using a human PPARγ cDNA probe labelled with [α-$^{32}$P] dCTP. The β-actin level shown in the lower lane is to demonstrate that equivalent amounts of RNA were loaded on each lane. (**C**) PPARγ protein expression in human gastric cancer cell lines. Approximately 50 μg of protein from each cell line was separated on an SDS-PAGE gel, probed with an anti-PPARγ antibody, and visualized with enhanced chemiluminescence. As an internal control, β-actin was detected weaker than that of troglitazone. Furthermore, we tested the effect of simultaneous administration of ligands of PPARγ and RXRα on the proliferation of gastric cancer cells. As shown in Figure 4, 9-cis RA augmented the growth inhibitory effect of troglitazone on gastric cancer cells. The experiment was also done on other cell lines and as the result was similar and there was no difference from PPARγ ligand response, detailed data are not shown.

### Changes of cell cycle profile by treatment with troglitazone

To investigate the mechanism of growth inhibition by PPARγ activation, we performed flow cytometric analysis to test the effect of troglitazone on the cell cycle profile. Representative cell cycle profiles are shown in Figure 5. Troglitazone-treated MKN-28, MKN-45 and AGS cells exhibited a significant increase in G1 phase associated with a decrease in S phase. On the other hand, G1 phase decreased slightly with an S phase increase in MKN-7. These results suggest that activation of PPARγ usually inhibits most cellular growth via induction of cell cycle arrest in G1,

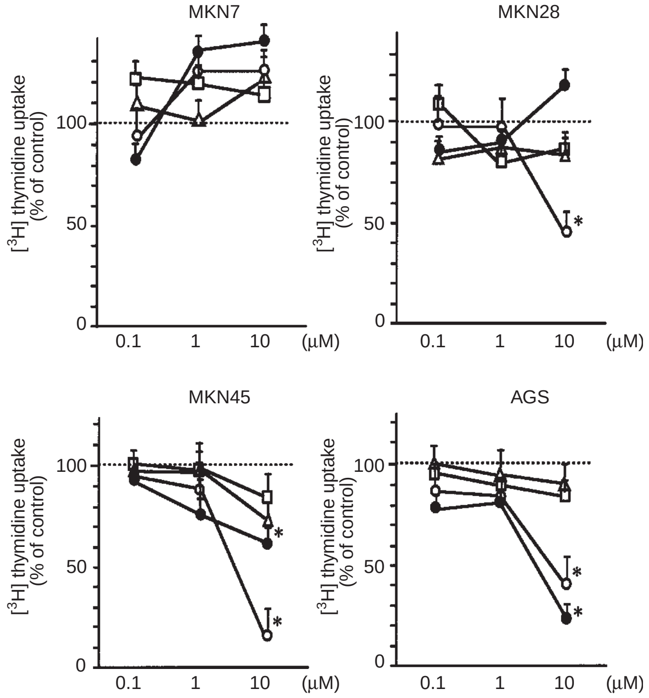

**Figure 3** Dose-dependent effect of troglitazone and 15d-PGJ2 on growth inhibition in human gastric cancer cell lines. Cells were seeded into 24-well plates at a density of 1 × 10$^5$ cells well$^{-1}$ and treated with troglitazone, 9-cis RA, indomethacin and 15d-PGJ2. After culture for 48 h, DNA synthesis was measured as [$^3$H] thymidine incorporation. Results are expressed as a percentage of control and each point represents the mean ± SE of three independent experiments. *$P < 0.05$ compared with untreated control. ○ = troglitazone, □ = 9-cis RA, △ = indomethacin, ● = 15d-PGJ2

however, in some cell lines such as MKN-7, they may induce a proliferative response.

### Detection of apoptosis in human gastric cancer cells

We evaluated whether PPARγ ligands induce apoptosis in human gastric cancer cells. Flow cytometric analysis was used to quantify apoptosis of cells treated with troglitazone 10 μM for 48 h. Treatment with troglitazone resulted in an increase of annexin V-positive cells in MKN-28, MKN-45, and AGS cells, but no increase in MKN-7 cells (Figure 6). As shown in Figure 7, we also confirmed morphologically the presence of apoptotic cells with condensed or fragmented nuclei by staining with Hoechst 33258.

### DISCUSSION

PPARγ, a subtype of the PPAR family, is predominantly expressed in adipose tissue, where it controls critical steps of lipid homeostasis and functions as a key trigger of adipocyte differentiation. PPARγ expression has also been found in cells from various lineages, such as liposarcoma, human breast cancer, colon cancer, and prostate cancer. Administration of PPARγ ligands was shown to inhibit the growth of these cells. In addition, a recent report demonstrated that in patients with advanced liposarcoma, troglitazone induced histological and biochemical differentiation of tumour cells to adipocytes (Demetri et al, 1999). These observations suggest that induction of terminal differentiation with PPARγ agonists may represent a novel therapeutic approach to human

© 2000 Cancer Research Campaign

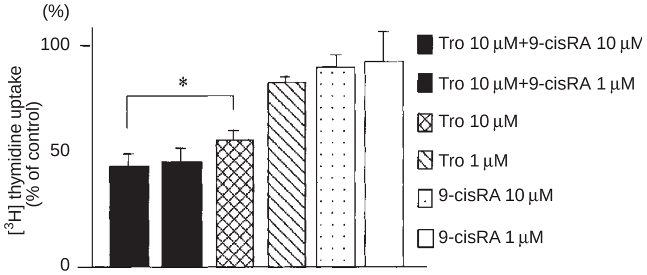

**Figure 4** Effect of simultaneous treatment with troglitazone (TRO) and 9-cis RA on growth inhibition. AGS cells were seeded ($1 \times 10^5$ cells well$^{-1}$) and treated with troglitazone and/or 9-cis RA. After culture for 48 h, [$^3$H] thymidine incorporation was measured. Results are expressed as a percentage of control. **P* < 0.05 compared with untreated control

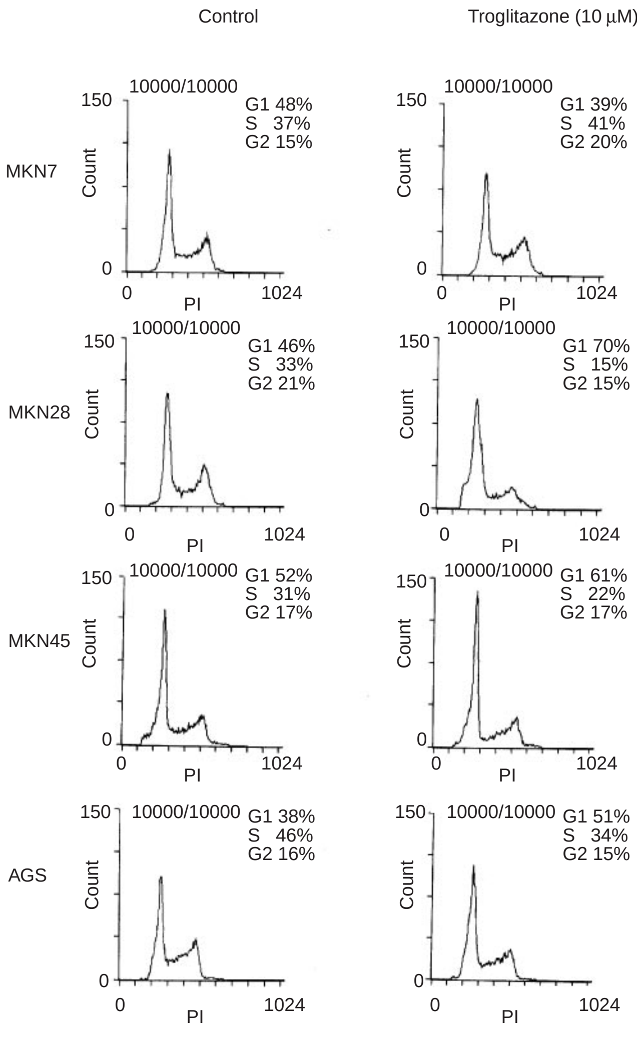

**Figure 5** Effect of a PPARγ ligand on cell cycle profile. Cells were cultured in presence of troglitazone (10 μM) or DMSO for 48 h, then harvested, fixed, stained with PI and analysed by flow cytometry. The values represent the number of cells in a phase of the cell cycle as a percentage of total cells malignancies. However, in colon cancer cells, the growth suppressing effect of PPARγ agonists, which was clearly shown in in vitro studies, was not necessarily confirmed by in vivo studies. Several reports have indicated that activation of PPARγ promotes

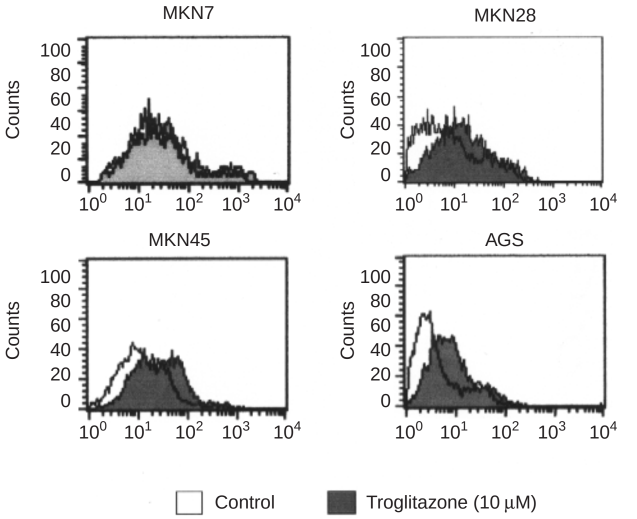

**Figure 6** Effect of a PPARγ ligand on apoptosis of gastric cancer cells. Cells were incubated in presence of troglitazone (10 μM) or DMSO for 48 h, then harvested and incubated with FITC-conjugated annexin V antibody and PI. Cells were analysed by flow cytometry; PI-negative and annexin V-positive cells were considered as early apoptotic cells the development of colon tumours, not only in C57BL/6J-APCmin/+ mice, a clinically relevant model for human familial adenomatous polyposis, but also in animal models that develop sporadic colon cancer (Lefebvre et al, 1998; Saez et al, 1998).

The expression of PPARγ in human gastric cancer has not been fully elucidated. We found strong expression of PPARγ in surgically resected human gastric cancer specimens irrespective of the differentiation of the cancer tissue. Furthermore, gastric antral mucosa with intestinal metaplasia was also shown to express PPARγ protein. This is the first report showing the presence of PPARγ not only in gastric cancer but also in non-cancerous mucosa of human stomach. In addition, we demonstrated an antiproliferative effect of PPARγ ligands in vitro. A significant growth inhibitory effect of troglitazone or 15d-PGJ2 was observed only in moderately and poorly differentiated adenocarcinoma cell lines. Conversely, growth inhibition by these ligands was not found in a well differentiated adenocarcinoma cell line, MKN-7, even at higher concentrations. Therefore, the role of PPARγ in growth control of human gastric cancer cells may depend on cellular differentiation, and well differentiated cancers may lose their sensitivity to growth control by PPARγ.

In this study, AGS cell line with low level PPARγ protein expression showed a significant antiproliferative effect, but MKN7 cells with high PPARγ expression did not. It seems difficult to explain the discrepancy between quantity of PPARγ protein expression and the anti-proliferative effect induced by PPARγ agonists on gastric carcinoma cell lines. Due to involvement of multiple factors like RXRα, cofactors, hsp70, etc, in PPARγ activation and binding to PPARγ-responsive element (PPRE), PPARγ agonist-mediated response may not depend only on the quantity of PPARγ protein present in a cell line. One possible explanation is PPARγ mutation in MKN7. Defect in other factors necessary for PPARγ activation and binding to PPARγ-responsive element (PPRE), or defect in the target gene(s) after PPARγ activation should also be considered as another possibility for ineffective response in MKN-7.

*© 2000 Cancer Research Campaign*

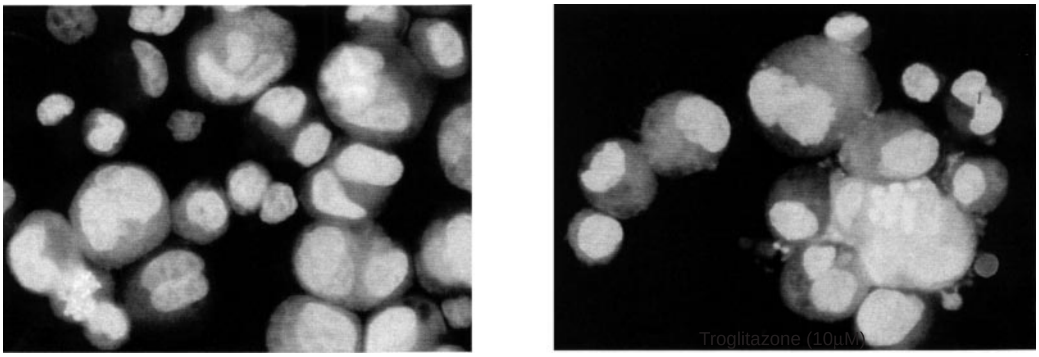

Control Troglitazone (10 μM)

**Figure 7** Appearance of AGS cells with nuclear condensation and fragmentation after treatment with troglitazone for 48 h. Cells were treated with troglitazone (10 μM) or DMSO for 48 h, the medium was removed, and the cells were fixed with 1.0% glutaraldehyde, stained with Hoechst 33258 dye and fluorescence visualized using a fluorescent microscope. The photomicrographs were taken at × 400 magnification.

Although the mechanism of growth inhibition via PPARγ in human gastric cancer cells has not been fully elucidated, it may be connected to the cell cycle, as reported with other mammalian cells. In our study, G1 cell cycle arrest was observed in moderately differentiated MKN-28 cells and in poorly differentiated AGS and MKN-45 cells after treatment with troglitazone. These results are comparable with that of colon cancer cells (Brockman et al, 1998; Kitamura et al, 1999). Therefore, growth inhibition by PPARγ ligands may be, at least in part, related to cell cycle arrest at G1 phase. In MKN-7 cells, cell cycle analysis after troglitazone treatment showed an increase in S phase with decreased G1 phase. Increased S phase in MKN-7 is also compatible with the [$^3$H] thymidine incorporation result of MKN-7 which show an increase too.

To investigate other possible mechanisms of the antiproliferative effect of PPARγ activation, the effect of PPARγ ligands on cellular apoptosis was studied in gastric cancer cells. PPARγ-induced apoptosis has recently been demonstrated in human breast cancer (Elstner et al, 1998; Clay et al, 1999), choriocarcinoma (Keelan et al, 1999), prostate cancer (Kubota et al, 1998) and endothelial cells (Bishop-Bailey and Hla, 1999). In the present study, treatment with troglitazone resulted in an increase in the number of apoptotic cells. Thus, apoptosis and cell cycle arrest are possible mechanisms for the growth inhibitory effect of PPARγ activation.

Recent reports have indicated that PPARγ ligands also suppress the clonal growth of leukaemia cell lines (Asou et al, 1999; Hirase et al, 1999; Sugimura et al, 1999). In fact, differentiation therapy with all-*trans* retinoic acid, a ligand for RARα, has already become one of the standard treatments for acute promyelocytic leukaemia (APML) (Huang et al, 1998; Warrell et al, 1991). Therapy that induces apoptosis and differentiation has recently been considered as a possible alternative treatment for various neoplastic diseases other than APML.

In the present study, we have shown that activation of one of the DNA-binding nuclear receptors PPARγ or RXRα has a growth suppressing effect on certain poorly differentiated gastric cancer cells. Poorly differentiated gastric cancer is frequently observed and it is the most lethal malignant neoplasm in several countries, including Japan. Therefore, therapy with potent PPARγ agonists may be a promising future approach for the treatment of poorly differentiated gastric cancer. Further studies will be necessary before PPARγ ligands can be used in patients with gastric cancer, but this nuclear receptor may provide a novel target for the treatment of gastric cancer in humans.

### REFERENCES

Asou H, Verbeek W, Williamson E, Elstner E, Kubota T, Kamada N and Koeffler HP (1999) Growth inhibition of myeloid leukemia cells by troglitazone, a ligand for peroxisome proliferator activated receptor gamma, and retinoids. *Int J Oncol* **15**: 1027–1031

Bishop-Bailey D and Hla T (1999) Endothelial cell apoptosis induced by the peroxisome proliferator-activated receptor (PPAR) ligand 15-deoxy-$\Delta^{12,14}$-prostaglandin J2. *J Biol Chem* **24**: 17042–17048

Brockman JA, Gupta RA and Dubois RN (1998) Activation of PPARγ leads to inhibition of anchorage-independent growth of human colorectal cancer cells. *Gastroenterology* **115**: 1049–1055

Chawla A, Schwarz EJ, Dimaculangan DD and Lazar MA (1994) Peroxisome proliferator-activated receptor (PPAR) γ: adipose-predominant expression and induction early in adipocyte differentiation. *Endocrinology* **135**: 798–800

Clay CE, Namen AW, Atsumi G, Willingham MC, High KP, Kute TE, Trimboli AJ, Fonteh AN, Dawson PA and Chilton FH (1999) Influence of J series prostaglandins on apoptosis and tumorigenesis of breast cancer cells. *Carcinogenesis* **20**: 1905–1911

Demetri GD, Fletcher CDM, Mueller E, Sarraf P, Naujoks RN, Campbell N, Spiegelman BM and Singer S (1999) Induction of solid tumor differentiation by the peroxisome proliferator-activated receptor γ ligand troglitazone in patients with liposarcoma. *Proc Natl Acad Sci USA* **96**: 3951–3956

Elstner E, Muller C, Koshizuka K, Asou H, Williamson EA, Park D, Shintaku P, Said JW, Heber D and Koeffler HP (1998) Ligands for peroxisome proliferator-activated receptor γ and retinoic acid receptor inhibit growth and induce apoptosis of human breast cancer cells in vitro and in BNX mice. *Proc Natl Acad Sci USA* **95**: 8806–8811

Forman BM, Tontonoz P, Chen J, Brun RP, Spiegelman BM and Evans RM (1995) 15-Deoxy-$\Delta^{12,14}$-prostaglandin J2 is a ligand for the adipocyte differentiation factor PPARγ. *Cell* **83**: 803–812

Hirase N, Yanase T, Mu YM, Muta K, Umemura T, Takayanagi R and Nawata H (1999) Thiazolidinedione induces apoptosis and monocytic differentiation in the promyelocytic leukemia cell line HL60. *Oncology* **57**(Suppl 2): 17–25

Huang ME, Ye YC, Chen SR, Chai JR, Lu JX, Zhoa L, Gu LJ and Wang ZY (1988) Use of all-trans retinoic acid in the treatment of acute promyelocytic leukemia. *Blood* **72**: 567–572

Hu E, Tontonoz P and Spiegelman BM (1995) Transdifferentiation of myoblasts by the adipogenic transcription factors PPARγ and C/EBPa. *Proc Natl Acad Sci USA* **92**: 9856–9860

Keelan JA, Sato TA, Marvin KW, Lander J, Gilmour RS and Mitchell MD (1999) 15-Dexoy-$\Delta^{12,14}$-prostaglandin J2, a ligand for peroxisome proliferator-activated receptor γ, induces apoptosis in JEG3 choriocarcinoma cells. *Biochem Biophys Res Commun* **262**: 579–585

Kitamura S, Miyazaki Y, Shinomura Y, Kondo S, Kanayama S and Mastuzawa Y (1999) Peroxisome proliferator-activated receptor γ induces growth arrest and differentiation markers of human colon cancers. *Jpn J Cancer Res* **90**: 75–80

Kliewer SA, Lenhard JM, Willson TM, Patel I, Morris DC and Lehmann JM (1995) A prostaglandin J2 metabolite binds peroxisome proliferator-activated receptor γ and promotes adipocyte differentiation. *Cell* **83**: 813–819

Kubota T, Koshizuka K, Asou H, Williamson EA, Said JW, Holded S, Miyoshi I and Koeffler HP (1998) Ligand for peroxisome proliferator-activated receptor γ (troglitazone) has potent antitumor effect against human prostate cancer both in vitro and in vivo. *Cancer Res* **58**: 3344–3352

Lefebvre AM, Chen I, Desreumaux P, Najib J, Fruchart JC, Geboes K, Briggs M, Heyman R and Auwerx J (1998) Activation of the peroxisome proliferator-activated receptor γ promotes the development of colon tumors in C57BL/6J-APCmin/+mice. *Nat Med* **4**: 1053–1057

Lehmann JM, Lenhard JM, Oliver BB, Ringold GM and Kliewer SA (1997) Peroxisome proliferator-activated receptor α and γ are activated by indomethacin and other non-steroidal anti-inflammatory drugs. *J Biol Chem* **272**: 3406–3410

Mukherjee R, Davles PJA, Cromble DL, Bischoff ED, Cesario RM, Jow L, Hamann LG, Boehm MF, Mondon CE, Nadzan AM, Paternitl JRJR and Heyman RA (1997) Sensitization of diabetic and obese mice to insulin by retinoid X receptor agonists. *Nature* **386**: 407–410

Pelton PD, Zhou L, Demarest KT and Burris TP (1999) PPARγ activation induces the expression of the adipocyte fatty acid binding protein gene in human monocytes. *Biochem Biophys Res Commun* **261**: 456–458

Saez E, Tontonoz P, Nelson MC, Alvarez JGA, Ming U-T, Baird SM, Thomazy VA and Evans RM (1998) Activators of the nuclear receptor PPARγ enhance colon polyp formation. *Nat Med* **4**: 1058–1061

Sarraf P, Mueller E, Jones D, King FJ, Deangelo DJ, Partridge JB, Holden SA, Chen LB, Singer S, Fletcher C and Spiegelman BM (1998) Differentiation and reversal of malignant changes in colon cancer through PPARγ. *Nat Med* **4**: 1046–1052

Sugimura A, Kiriyama Y, Nochi H, Tsuchiya H, Tamoto K, Sakurada Y, Ui M and Tokumitsu Y (1999) Troglitazone suppresses cell growth of myeloid leukemia cell lines by induction of p21 Waf1/Cip1 cyclin-dependent kinase inhibitor. *Biochem Biophys Res Commun* **261**: 833–837

Takahashi N, Okumura T, Motomura W, Fujimoto Y, Kawabata I and Kohgo Y (1999) Activation of PPARγ inhibits cell growth and apoptosis in human gastric cancer cells. *FEBS Lett* **455**: 135–139

Tontonoz P, Hu E and Spiegelman BM (1994) Stimulation of adipogenesis in fibroblasts by PPARγ 2, a lipid-activated transcription factor. *Cell* **79**: 1147–1156

Tontonoz P, Singer S, Forman AM, Sarraf P, Fletcher JA, Fletcher CDM, Brun RP, Mueller E, Altiok S, Oppenheim H, Evans RM and Spiegelman BM (1997). Terminal differentiation of human liposarcoma cells induced by ligands for peroxisome proliferator-activated receptor γ and the retinoid X receptor. *Proc Natl Acad Sci USA* **94**: 237–241

Tontonoz P, Nagy L, Alvarez JGA, Thomazy VA and Evans RM (1998) PPARγ promotes monocyte/macrophage differentiation and uptake of oxidized LDL. *Cell* **93**: 241–252

Warrell RP, Frankel SR, Miller WHJR, Scheinberg DA, Itri LM, Hittelman WN, Vyas R, Andreeff M, Tafuri A, Jakubowski A, Gabrilove J, Gordon MS and Dmitrovsky E (1991) Differentiation therapy of acute promyelocytic leukemia with tretinoin (all-trans-retinoic acid). *N Engl J Med* **324**: 1385–1393

 *© 2000 Cancer Research Campaign*
